# Explainable Meta-Learning Ensemble Framework for Predicting Insulin Dose Adjustments in Diabetic Patients: A Comparative Machine Learning Approach with SHAP-Based Clinical Interpretability

**DOI:** 10.3390/medicina62030502

**Published:** 2026-03-09

**Authors:** Emek Guldogan, Burak Yagin, Hasan Ucuzal, Abdulmohsen Algarni, Fahaid Al-Hashem, Mohammadreza Aghaei

**Affiliations:** 1 Department of Biostatistics and Medical Informatics, Faculty of Medicine, Inonu University, Malatya 44280, Türkiye; 2 Department of Computer Science, King Khalid University, Abha 61421, Saudi Arabia; 3 Department of Physiology, College of Medicine, King Khalid University, Abha 61421, Saudi Arabia; 4 Department of Ocean Operations and Civil Engineering, Norwegian University of Science and Technology (NTNU), 6009 Alesund, Norway

**Keywords:** machine learning, ensemble methods, insulin dose prediction, diabetes mellitus, explainable artificial intelligence, SHAP, LIME, clinical decision support, meta-learning, gradient boosting

## Abstract

*Background and Objectives*: Diabetes mellitus represents one of the most prevalent chronic metabolic disorders worldwide, necessitating precise insulin dose management to prevent both acute and long-term complications. The optimization of insulin dosing remains a significant clinical challenge, as inappropriate dosing can lead to hypoglycemia or hyperglycemia, each carrying substantial morbidity risks. Machine learning approaches have emerged as promising tools for developing clinical decision support systems; however, their practical implementation requires both high predictive accuracy and model interpretability. This study aimed to develop and evaluate an explainable machine learning framework for predicting insulin dose adjustments in diabetic patients. We sought to compare multiple ensemble learning approaches and identify the optimal model configuration that balances predictive performance with clinical interpretability through comprehensive SHAP and LIME analyses. *Materials and Methods*: A comprehensive dataset comprising 10,000 patient records with 12 clinical and demographic features was utilized. We implemented and compared nine machine learning models, including gradient boosting variants (XGBoost, LightGBM, CatBoost, GradientBoosting), AdaBoost, and four ensemble strategies (Voting, Stacking, Blending, and Meta-Learning). Model interpretability was achieved through SHapley Additive exPlanations (SHAP) and Local Interpretable Model-agnostic Explanations (LIME) analyses. Performance was evaluated using accuracy, weighted F1-score, area under the receiver operating characteristic curve (AUC-ROC), precision-recall AUC (PR-AUC), sensitivity, specificity, and cross-entropy loss. *Results*: The Meta-Learning Ensemble achieved superior performance across all evaluation metrics, attaining an accuracy of 81.35%, weighted F1-score of 0.8121, macro-averaged AUC-ROC of 0.9637, and PR-AUC of 0.9317. The model demonstrated exceptional sensitivity (86.61%) and specificity (91.79%), with particularly high performance in detecting dose reduction requirements (100% sensitivity for the ‘down’ class). SHAP analysis revealed insulin sensitivity, previous medications, sleep hours, weight, and body mass index as the most influential predictors across different insulin adjustment categories. The meta-model feature importance analysis indicated that LightGBM probability estimates contributed most significantly to the ensemble predictions. *Conclusions*: The proposed explainable Meta-Learning Ensemble framework demonstrates robust predictive capability for insulin dose adjustment recommendations while maintaining clinical interpretability. The integration of SHAP-based explanations facilitates clinician understanding of model predictions, supporting transparent and informed decision-making in diabetes management. This approach represents a significant advancement toward the clinical implementation of artificial intelligence in personalized insulin therapy.

## 1. Introduction

Diabetes mellitus has emerged as one of the most pressing global health challenges of the twenty-first century, affecting approximately 537 million adults worldwide as of 2021, with projections indicating a rise to 643 million by 2030, and 783 million by 2045 [1]. This chronic metabolic disorder is characterized by persistent hyperglycemia resulting from defects in insulin secretion, insulin action, or both, leading to progressive damage across multiple organ systems including cardiovascular, renal, neurological, and ophthalmic complications. The global burden of diabetes extends beyond direct health impacts, imposing substantial economic costs through healthcare expenditures, productivity losses, and long-term disability management [2,3,4].

The management of diabetes mellitus fundamentally relies on achieving and maintaining optimal glycemic control, which frequently necessitates insulin therapy, particularly in patients with Type 1 diabetes and advanced Type 2 diabetes. However, the determination of appropriate insulin dosing remains a complex clinical challenge, requiring careful consideration of numerous patient-specific factors including body weight, glycemic patterns, dietary intake, physical activity levels, concurrent medications, and individual metabolic variability. Suboptimal insulin dosing carries significant clinical consequences: insufficient dosing leads to persistent hyperglycemia and accelerated development of microvascular and macrovascular complications, while excessive dosing precipitates hypoglycemic episodes that range from mild cognitive impairment to life-threatening seizures and cardiac arrhythmias [5,6].

Traditional approaches to insulin dose adjustment have relied predominantly on clinical algorithms, physician experience, and patient self-monitoring. While these methods have formed the foundation of diabetes care for decades, they inherently suffer from limitations including inter-provider variability, delayed responsiveness to changing patient conditions, and the inability to simultaneously integrate and process multiple predictive variables. The advent of continuous glucose monitoring (CGM) technology and electronic health records has generated unprecedented volumes of patient data, creating both an opportunity and a necessity for more sophisticated analytical approaches [7,8].

Machine learning (ML) has demonstrated remarkable potential in addressing the aforementioned challenges by identifying complex, non-linear patterns within multidimensional clinical datasets that may elude traditional statistical methods [9,10,11].

Recent studies have successfully applied various ML algorithms to diabetes-related prediction tasks, including blood glucose forecasting, hypoglycemia risk assessment, and treatment response prediction [12]. Ensemble learning methods, which combine predictions from multiple base models to achieve superior generalization performance, have shown particular promise in healthcare applications (e.g., improved robustness across patient cohorts or enhanced predictive stability) due to their ability to reduce overfitting while capturing diverse aspects of the underlying data distribution [13,14].

Gradient boosting algorithms, including XGBoost, LightGBM, and CatBoost, have emerged as particularly effective approaches for tabular clinical data, consistently achieving state-of-the-art performance across diverse medical prediction tasks [15,16,17,18].

These algorithms handle heterogeneous clinical features, capture non-linear interactions, and incorporate built-in regularization, making them particularly effective for tabular medical data [19,20]. Recent comparative studies have demonstrated that gradient boosting methods frequently outperform traditional ML classifiers in diabetes-related classification problems [21,22].

Despite these advances, the clinical adoption of ML-based decision support systems has been hampered by concerns regarding model interpretability and transparency [23]. Healthcare practitioners rightfully require not merely accurate predictions but also comprehensible explanations that align with clinical reasoning and support informed decision-making. This imperative has catalyzed the emergence of explainable artificial intelligence (XAI) as a critical component of medical ML applications, with techniques such as SHapley Additive exPlanations (SHAP) and Local Interpretable Model-agnostic Explanations (LIME) enabling the decomposition of complex model outputs into interpretable feature contributions [24,25]. Nevertheless, these techniques have inherent limitations: SHAP analysis incurs considerable computational costs, particularly with model-agnostic implementations, while LIMEs may exhibit instability due to stochastic perturbation sampling [26]. Despite these constraints, their complementary application remains the most robust approach for achieving clinically meaningful model transparency. The integration of SHAP and LIME with ensemble learning enables complementary global and local model explanations, supporting clinician trust and transparent decision-making [27].

The present study addresses these considerations by developing and validating an explainable meta-learning ensemble framework for predicting insulin dose adjustments in diabetic patients. Our approach synthesizes the predictive power of multiple gradient boosting algorithms with advanced ensemble strategies while incorporating comprehensive interpretability analysis. Specifically, we aimed to: (1) compare the performance of multiple ML algorithms and ensemble configurations for multi-class insulin dose adjustment prediction; (2) identify the optimal model architecture that maximizes both accuracy and clinical utility; (3) elucidate the relative importance and contribution patterns of clinical features through SHAP and LIME analyses; and (4) develop a transparent, clinically actionable framework suitable for integration into diabetes management workflows.

The main contributions of this study can be summarized as follows:

1. We propose a novel explainable meta-learning ensemble framework that synergistically combines multiple gradient boosting algorithms (XGBoost, LightGBM, CatBoost, GradientBoosting) through a 5-fold cross-validation-based meta-feature generation strategy, achieving superior performance compared to individual classifiers and conventional ensemble methods.
2. We provide a comprehensive comparative analysis of nine machine learning models for multi-class insulin dose adjustment prediction—a more clinically nuanced task than binary diabetes detection—offering practical guidance for algorithm selection in similar clinical applications.
3. We integrate dual-layer explainability through both SHAP (global interpretability) and LIME (local interpretability) analyses, enabling clinicians to understand population-level feature importance patterns and individual patient-specific prediction rationales simultaneously.

## 2. Materials and Methods

### 2.1. Study Design and Data Source

This study utilized the publicly available “Insulin Dosage” dataset obtained from the Kaggle platform (https://www.kaggle.com). The dataset comprises 10,000 patient records with 12 clinical and demographic variables related to insulin dose adjustment decisions. The dataset encompasses a comprehensive range of clinical, demographic, and metabolic parameters, including age, gender, family history of diabetes, fasting glucose levels, glycated hemoglobin (HbA1c), body mass index (BMI), body weight, physical activity levels, and insulin sensitivity indices, among others. It was specifically curated to support the development and validation of insulin dose adjustment prediction models, with each patient encounter containing a labeled outcome indicating the recommended insulin modification [26]. Given its structured design and clinically relevant feature set, this dataset provides a robust foundation for developing predictive models aimed at optimizing insulin dosage management in diabetic populations [28].

Regarding ethical considerations, this study exclusively utilized a de-identified, publicly available dataset with no direct patient contact or intervention; therefore, formal institutional review board (IRB) approval was deemed exempt. The dataset contains no personally identifiable information, and its public release on the Kaggle platform for research purposes was authorized by the data contributor. This approach aligns with established ethical guidelines for secondary analysis of publicly available, anonymized data, which typically exempt such studies from formal ethical review requirements [29].

The study adhered to established guidelines for the development and reporting of clinical prediction models, including the Transparent Reporting of a Multivariable Prediction Model for Individual Prognosis or Diagnosis (TRIPOD) statement as extended for artificial intelligence applications [30].

### 2.2. Study Population and Variables

Twelve predictor variables were extracted and analyzed, selected based on their established clinical relevance to insulin dosing decisions and alignment with current diabetes management guidelines [2]. The variable selection was informed by three complementary criteria: (1) physiological relevance to insulin pharmacodynamics and glucose homeostasis, (2) routine availability in standard clinical practice, and (3) prior evidence from the diabetes prediction literature. Demographic characteristics included gender and age, as both factors significantly influence insulin sensitivity, with age-related decline in beta-cell function and sex-specific differences in body composition affecting insulin requirements. Metabolic indicators comprised glucose level, HbA1c, BMI, weight, insulin sensitivity, and creatinine. Glucose level and HbA1c serve as primary indicators of glycemic control, directly informing dose titration decisions. BMI and weight are critical determinants of insulin dosing, as adiposity correlates inversely with insulin sensitivity, often necessitating higher doses in obese patients [6]. Insulin sensitivity was included as a direct measure of individual responsiveness to exogenous insulin administration. Creatinine was incorporated to reflect renal function status, which profoundly impacts insulin clearance and hypoglycemia risk, as reduced renal function prolongs insulin half-life and necessitates dose reduction. Lifestyle factors encompassed physical activity, food intake, and sleep hours. Physical activity acutely enhances insulin sensitivity and glucose uptake, requiring corresponding dose adjustments to prevent hypoglycemia. Food intake directly influences postprandial glucose excursions and prandial insulin requirements. Sleep duration was included based on emerging evidence linking sleep disturbances to impaired glucose tolerance and altered insulin sensitivity through disruption of circadian metabolic regulation. Clinical history was represented by previous medications, capturing the therapeutic context and potential drug interactions that may modify insulin requirements, such as concurrent use of corticosteroids, beta-blockers, or other glucose-altering medications. Collectively, these twelve variables represent a clinically coherent and practically accessible feature set that balances comprehensiveness with real-world applicability in routine diabetes care settings.

The outcome variable was categorized into four distinct classes reflecting clinical insulin management decisions: (1) ‘no’ indicating no change in current insulin regimen (n = 701, 7.01%); (2) ‘down’ indicating a recommendation for dose reduction (n = 610, 6.10%); (3) ‘up’ indicating a recommendation for dose increase (n = 3363, 33.63%); and (4) ‘steady’ indicating maintenance of the current therapeutic approach with continued monitoring (n = 5326, 53.26%). This multi-class classification schema aligns with standard clinical practice paradigms for insulin titration.

It should be noted that the ‘no’ and ‘steady’ classes, whilst both representing the absence of a dose change, are clinically distinct. The ‘no’ class denotes a situation in which the patient’s glycemic profile is sufficiently stable to require neither a dose adjustment nor intensified follow-up in the immediate term. The ‘steady’ class, by contrast, indicates that the current regimen is maintained but that the patient’s clinical status warrants continued close monitoring, as glycemic parameters may be approaching a threshold at which a future adjustment could become necessary. Regarding the ‘down’ and ‘up’ classes, ongoing monitoring remains clinically necessary following any dose adjustment in order to evaluate the patient’s response to the change and to guide subsequent titration decisions. These class definitions are derived from the labelling schema of the Kaggle dataset used in this study and have not been independently verified against clinical titration guidelines or electronic health records.

Table 1 presents the feature operationalization summary, including the measurement unit, clinical definition, and dataset coding format for each predictor variable used in the model.

### 2.3. Data Preprocessing

Data preprocessing procedures were implemented to ensure data quality and optimize model performance. Missing value assessment revealed complete data across all variables, obviating the need for imputation procedures; this completeness reflects prior curation by the dataset contributor, as the publicly available Kaggle repository version was preprocessed to remove records with missing entries and ensure data integrity for machine learning applications. Feature standardization was performed using z-score normalization to ensure comparable scaling across variables with disparate measurement units and ranges. The dataset was partitioned using stratified random sampling to maintain class proportions across subsets, with an 80:20 split ratio yielding a training set of 8000 observations and a test set of 2000 observations. To prevent data leakage and ensure unbiased performance estimation, all preprocessing transformations including feature standardization were implemented within a scikit-learn Pipeline framework, whereby normalization parameters were computed exclusively from training data within each cross-validation fold and subsequently applied to the corresponding validation and test partitions, thereby ensuring that no information from held-out samples influenced the preprocessing stage [31].

### 2.4. Machine Learning Algorithms

A comprehensive suite of ML algorithms was evaluated, encompassing both individual classifiers and ensemble methods, with algorithm selection guided by three primary considerations: demonstrated effectiveness on tabular clinical data, complementary architectural characteristics to promote ensemble diversity, and computational feasibility for clinical deployment. Base classifiers included four gradient boosting variants—XGBoost (Extreme Gradient Boosting), LightGBM (Light Gradient Boosting Machine), GradientBoosting, and CatBoost—deliberately selected to capture algorithmic diversity despite their shared boosting paradigm, as each implements distinct tree construction strategies (histogram-based versus exact greedy), leaf growth patterns (leaf-wise versus level-wise), and categorical feature handling mechanisms (native encoding versus numerical transformation), thereby generating heterogeneous prediction patterns that enhance ensemble performance through error decorrelation [32]. AdaBoost was included as a representative adaptive boosting approach with fundamentally different sample weighting mechanics to further augment base learner diversity. Ensemble methods comprised Voting Ensemble employing soft voting with weighted probability averaging to leverage calibrated prediction confidences, Stacking Ensemble utilizing a two-layer architecture with logistic regression as the meta-learner—selected for its inherent regularization properties, probabilistic interpretability, and resistance to overfitting when combining correlated base model outputs [33], Blending Ensemble implementing a holdout-based approach with the top three performing models, and Meta-Learning Ensemble employing 5-fold cross-validation-based meta-feature generation with a gradient boosting meta-model to optimally learn non-linear combination weights from out-of-fold predictions. Model diversity within ensemble frameworks was explicitly ensured through three mechanisms: (1) inclusion of algorithmically distinct base learners with varying inductive biases, (2) hyperparameter configurations that promoted differential decision boundary formations, and (3) quantitative assessment of pairwise prediction disagreement rates to verify sufficient heterogeneity among constituent models, as ensemble effectiveness fundamentally depends on the errors of base learners being uncorrelated [34].

Hyperparameter optimization was conducted using RandomizedSearchCV with 5-fold stratified cross-validation, enabling efficient exploration of the hyperparameter space while balancing computational requirements. The optimization objective was weighted F1-score to appropriately account for class imbalance in the outcome variable. Table 2 presents the optimized hyperparameter configurations for each algorithm.

### 2.5. Model Pipeline

The pipeline implemented in this study was executed in the following sequence: (1) Data were split into 80% training/20% test sets; the test set was withheld from all subsequent steps. (2) All preprocessing (e.g., scaling, imputation) was applied within each cross-validation fold exclusively on the training set to prevent data leakage. (3) Hyperparameter optimization (RandomizedSearchCV) was performed exclusively on the training set via an inner 5-fold cross-validation loop; this cross-validation was used solely for hyperparameter selection and did not contribute to final performance estimation. (4) Using the optimized base models, meta-features were generated through 5-fold cross-validation on the training set; test set meta-features were derived separately by applying the fully trained base models to the held-out test set without any fold involvement. (5) Final evaluation was conducted solely on the held-out 20% test set (n = 2000), which was not involved in any prior stage. This sequential structure ensures strict separation between hyperparameter tuning, meta-feature generation, and final evaluation, with no data leakage across stages. To prevent data leakage, all operations were applied in the following order within the sklearn Pipeline framework.

Data split: The entire dataset was first partitioned into 80% training and 20% test sets. The test set was locked from this point forward and excluded from all intermediate steps.

Base learner hyperparameter optimization: Hyperparameter optimization was performed for each base model (XGBoost, LightGBM, AdaBoost, GradientBoosting, CatBoost) exclusively on the training set using RandomizedSearchCV with 5-fold stratified cross-validation as the inner tuning loop. This inner cross-validation was used solely for hyperparameter selection and did not serve as a performance estimation step. Standardization was incorporated into the Pipeline at this stage and fitted solely on training data within each fold to prevent data leakage.

Out-of-fold meta-feature generation: The optimized base models were retrained on the training set using 5-fold stratified cross-validation to generate out-of-fold predictions for each sample. These predictions served as the training data for the meta-learner. No test set data was involved at this stage.

Meta-learner training and optimization: The meta-learner was trained exclusively on the meta-features derived from out-of-fold predictions, and its hyperparameters were optimized solely on this data.

**Figure 1:**
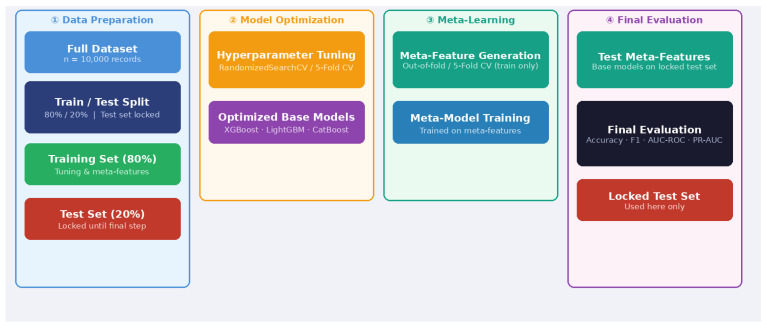
ML Pipeline: Hyperparameter Tuning + Meta-Learning. The pipeline consists of four sequential stages: (1) Data Preparation with 80/20 train-test split, (2) Model Optimization via RandomizedSearchCV with 5-fold inner CV for hyperparameter tuning only, (3) Meta-Learning with out-of-fold meta-feature generation and meta-model training, and (4) Final Evaluation on the locked test set.

Final evaluation on the held-out test set: Base models were retrained on the full training set and applied to the hold-out 20% test set (n = 2000) to generate test meta-features. The meta-learner was then evaluated on these features. All performance metrics reported including Accuracy, F1, AUC-ROC, PR-AUC, Sensitivity, Specificity, and Log Loss—were computed exclusively on the test set (Figure 1).

### 2.6. Model Evaluation Metrics

Comprehensive performance evaluation was conducted using multiple metrics to capture different aspects of classification performance. Multi-class AUC-ROC and PR-AUC were computed using the one-versus-rest (OvR) strategy, wherein each class was individually evaluated against all remaining classes combined, with final values obtained through macro-averaging across all four insulin adjustment classes to ensure equal weighting regardless of class prevalence. Accuracy was included as a conventional baseline metric to facilitate comparison with prior literature; however, given the inherent class imbalance in the dataset—ranging from 6.10% for the minority ‘down’ class to 53.26% for the majority ‘steady’ class—accuracy was interpreted cautiously as a supplementary rather than primary metric, alongside imbalance-robust measures including weighted F1-score, which accounts for class frequencies in aggregation, and macro-averaged AUC-ROC and PR-AUC, which provide threshold-independent discrimination assessment with equal consideration for minority classes. Cross-entropy loss, also termed log loss, was computed exclusively on the held-out test set to provide an unbiased evaluation of probability calibration quality without potential inflation from training data exposure; this metric quantifies the divergence between predicted probability distributions and actual class labels, with lower values indicating superior calibration.

### 2.7. Explainability Analysis

Model interpretability was established through two complementary approaches. SHAP analysis employed KernelExplainer rather than the computationally more efficient TreeExplainer, as the Meta-Learning Ensemble constitutes a heterogeneous stacked architecture wherein probability outputs from multiple base learners serve as meta-features for a secondary gradient boosting model; this composite structure precludes direct application of TreeExplainer, which requires access to homogeneous tree-based internal structures, whereas KernelExplainer provides model-agnostic Shapley value approximations applicable to arbitrary model architectures regardless of their internal complexity [35]. SHAP values were computed for a stratified random sample of 100 test instances, a sample size selected to balance computational tractability—given KernelExplainer’s exponential complexity—with statistical representativeness, ensuring proportional inclusion of all four insulin adjustment classes while exceeding recommended minimum thresholds for stable global feature importance estimation in multi-class settings [36]. LIME provided instance-specific feature contribution analysis, with cases systematically selected according to three criteria: (1) correctly classified instances with high prediction confidence (probability ≥ 0.90) representing typical decision patterns for each class, (2) borderline cases with moderate confidence to examine decision boundaries, and (3) clinically critical cases involving dose reduction recommendations where model transparency is paramount for hypoglycemia prevention and patient safety. To assess the clinical plausibility of the explainability outputs, SHAP and LIME feature importance patterns were evaluated against established pathophysiological mechanisms and published clinical guidelines for insulin dose titration. The prominence of insulin sensitivity as the leading predictor for dose increase recommendations and HbA1c for dose reduction decisions is consistent with accepted medical knowledge and standard diabetes management protocols, thereby supporting the biological plausibility of the predictive framework. Additional analyses for the best-performing model included meta-model feature importance assessment, ensemble diversity analysis, model calibration evaluation, and decision boundary visualization using principal component analysis (PCA) dimensionality reduction.

When computing SHAP values, the explainer type was determined separately according to the structure of each model: TreeExplainer, which offers superior computational efficiency and stability, was preferred for tree-based models such as XGBoost, LightGBM, CatBoost, and GradientBoosting. KernelExplainer was used only for models not supported by TreeExplainer. For KernelExplainer applications, the background dataset was constructed from 200 representative samples selected from the training set by random sampling and k-means clustering. SHAP values were computed on the full test set, and the stability of feature importance rankings was verified across different background sample sizes (50, 100, and 200).

The instances presented in the LIME analysis were selected through random sampling from each class, without any biased selection. However, in the revised analysis, the examination was not limited to random selection alone; misclassified cases and borderline instances in which the model’s predicted probability fell within the 0.4–0.6 range were also systematically examined. To assess explanation stability, the LIME analysis was repeated 10 times for each instance with different random seeds, and the consistency of feature rankings was measured.

### 2.8. Statistical Analysis and Software

All analyses were conducted using Python 3.11.14 (Python Software Foundation, Wilmington, NC, USA) with the following libraries: scikit-learn (v1.3) for base classifiers and evaluation metrics, XGBoost (v2.0), LightGBM (v4.0), and CatBoost (v1.2) for gradient boosting implementations, SHAP (v0.48) and LIME (v0.2) for interpretability analyses, and Pandas (v2.3), NumPy (v1.26), and Matplotlib (v3.9) for data manipulation and visualization.

## 3. Results

### 3.1. Dataset Characteristics

**Figure 2:**
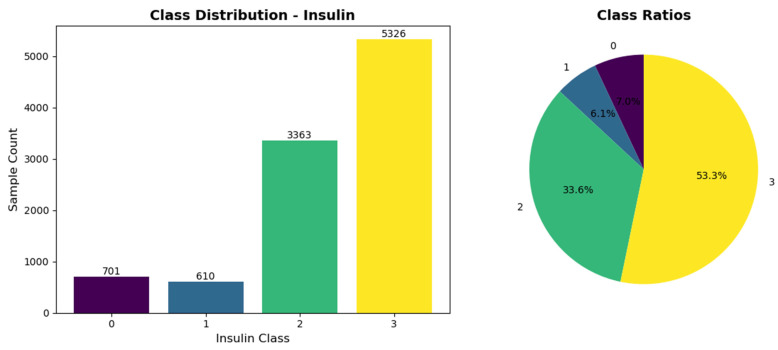
Distribution of insulin dose adjustment classes in the study population (N = 10,000). The four outcome categories represent clinical recommendations: ‘no’ (no change required; coded ‘0’), ‘down’ (dose reduction; coded ‘1’), ‘up’ (dose increase; coded ‘2’), and ‘steady’ (maintain current regimen with monitoring; coded ‘3’).

The analytical dataset comprised 10,000 patient records with complete data across all 12 predictor variables. The distribution of the outcome variable demonstrated notable class imbalance, with ‘steady’ representing the majority class (53.26%) and ‘down’ constituting the minority class (6.10%). Figure 2 illustrates the class distribution, reflecting typical clinical scenarios where most patients maintain stable insulin requirements while dose adjustments are recommended for a subset of patients.

### 3.2. Comparative Model Performance

**Figure 3:**
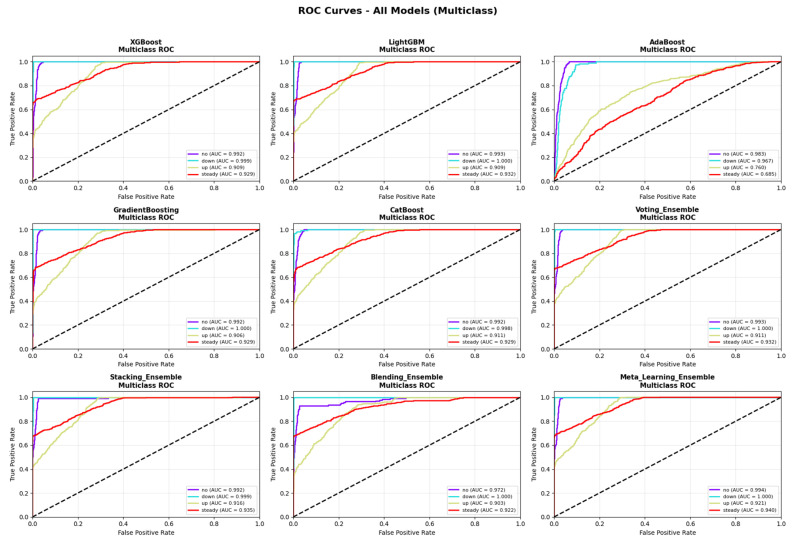
The ROC curves for all evaluated models, demonstrating the superior discriminative ability of the Meta-Learning Ensemble across all classification thresholds.

**Figure 4:**
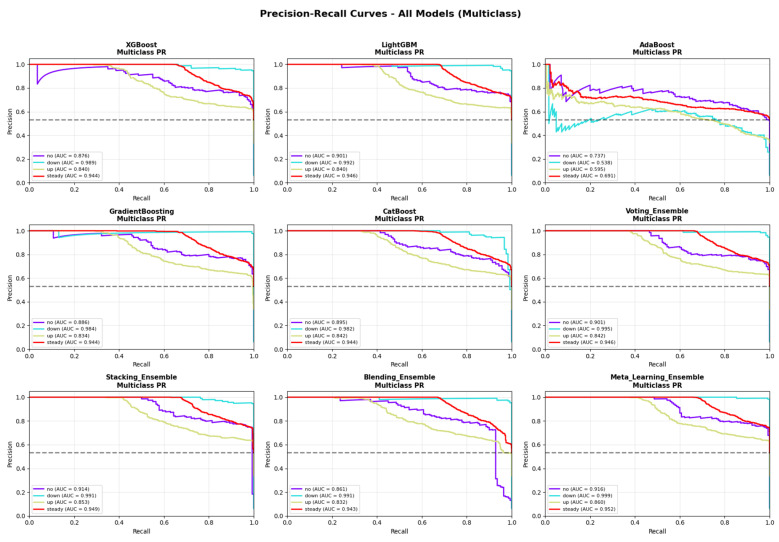
Precision-Recall (PR) curves for all evaluated machine learning models. PR curves are particularly informative for imbalanced classification problems. The Meta-Learning Ensemble achieves macro-averaged PR-AUC of 0.9317.

Table 3 presents the comprehensive performance comparison across all evaluated models. The Meta-Learning Ensemble achieved the highest performance on all primary evaluation metrics, with an accuracy of 81.35%, weighted F1-score of 0.8121, macro-averaged AUC-ROC of 0.9637, and PR-AUC of 0.9317. This model also demonstrated superior sensitivity (86.61%) and specificity (91.79%) compared to all other approaches, with the lowest cross-entropy loss (0.2939), indicating well-calibrated probability estimates. The ROC curves for all evaluated models are presented in Figure 3, demonstrating the superior discriminative ability of the Meta-Learning Ensemble across all classification thresholds. The corresponding PR curves (Figure 4) further confirm the model’s robust performance, particularly important given the class imbalance present in the dataset.

### 3.3. Class-Specific Performance Analysis

Table 4 presents the detailed class-specific performance metrics for the Meta-Learning Ensemble. The model demonstrated exceptional performance for the ‘dose reduction’ (down) class, achieving perfect sensitivity (100%) with high precision (98.39%), yielding an F1-score of 0.9919. Performance for the ‘no change’ class was similarly robust, with sensitivity of 91.43% and F1-score of 0.8449. The ‘steady’ class achieved balanced precision (83.83%) and recall (85.16%), while the ‘dose increase’ (up) class demonstrated the most challenging classification with sensitivity of 69.84%.

**Figure 5:**
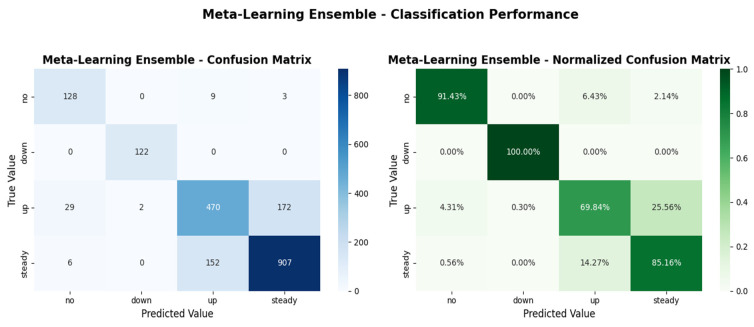
Confusion matrix for the Meta-Learning Ensemble on the test set (n = 2000). The diagonal elements represent correct classifications, while off-diagonal elements indicate misclassifications. The model demonstrates particularly strong performance for the ‘dose reduction’ class with no false negatives.

Figure 5 presents the confusion matrix for the Meta-Learning Ensemble, visualizing the classification patterns across all four insulin adjustment classes.

**Figure 6:**
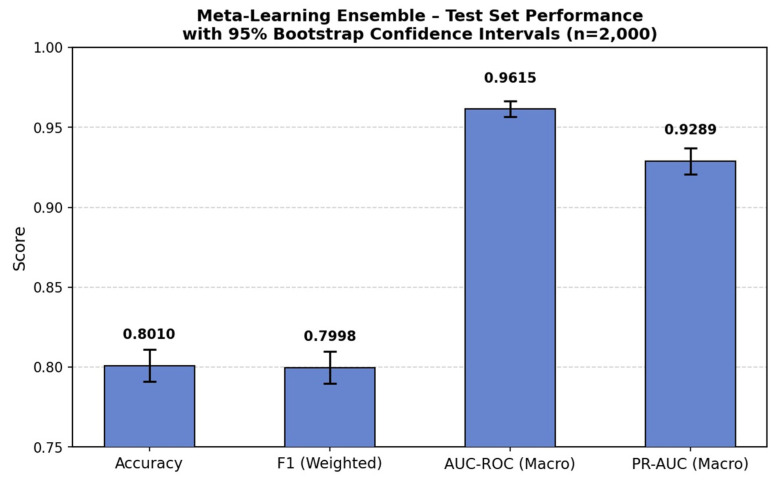
Test set performance metrics of the Meta-Learning Ensemble with 95% bootstrap confidence intervals (n = 2000 resamples). Error bars indicate the lower and upper bounds of the confidence interval for each metric.

**Figure 7:**
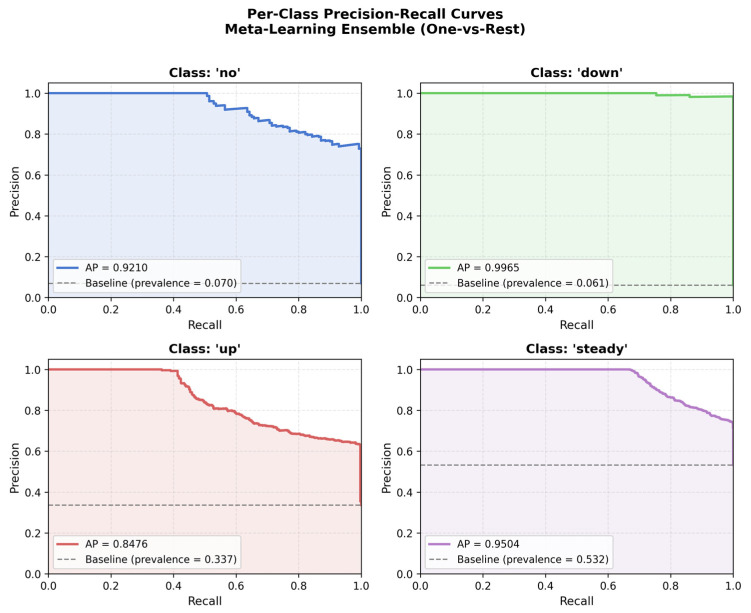
Per-class Precision-Recall curves for the Meta-Learning Ensemble using one-versus-rest evaluation. The dashed horizontal line indicates the baseline prevalence for each class. Average Precision (AP) values are reported in the legend of each panel.

Additional statistical and diagnostic analyses were conducted for the Meta-Learning Ensemble. Bootstrap confidence intervals (n = 2000 resamples) for held-out test performance are presented in Figure 6; the 95% confidence intervals were: Accuracy 0.8010 [0.7835–0.8180], weighted F1-score 0.7998 [0.7824–0.8169], macro-averaged AUC-ROC 0.9615 [0.9563–0.9663], and macro-averaged PR-AUC 0.9289 [0.9169–0.9391]. These narrow intervals indicate stable and consistent performance on the held-out test set. Per-class Precision-Recall curves are presented in Figure 7, with class-wise Average Precision (AP) values of 0.9210 for the ‘no’ class, 0.9965 for ‘down’, 0.8476 for ‘up’, and 0.9504 for ‘steady’. The lowest AP was observed for the ‘up’ class (AP = 0.8476), consistent with the lower sensitivity reported in the confusion matrix analysis.

### 3.4. SHAP Analysis and Feature Importance

**Figure 8:**
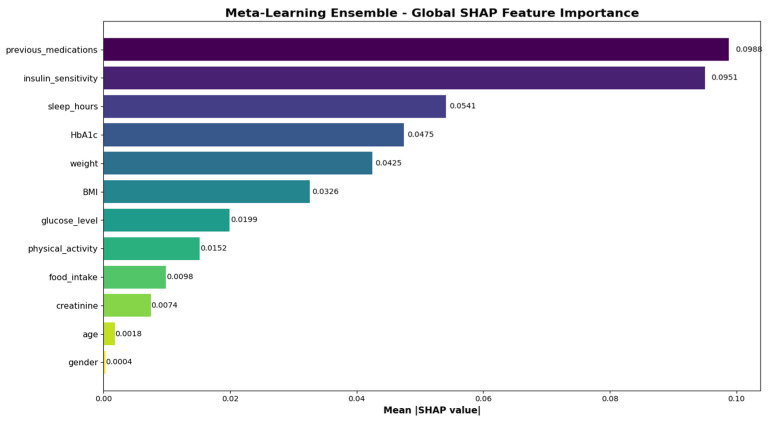
Global SHAP feature importance for the Meta-Learning Ensemble. Bar lengths represent mean absolute SHAP values across all test samples and classes. Insulin sensitivity, previous medications, and sleep hours emerge as the top three predictors.

**Figure 9:**
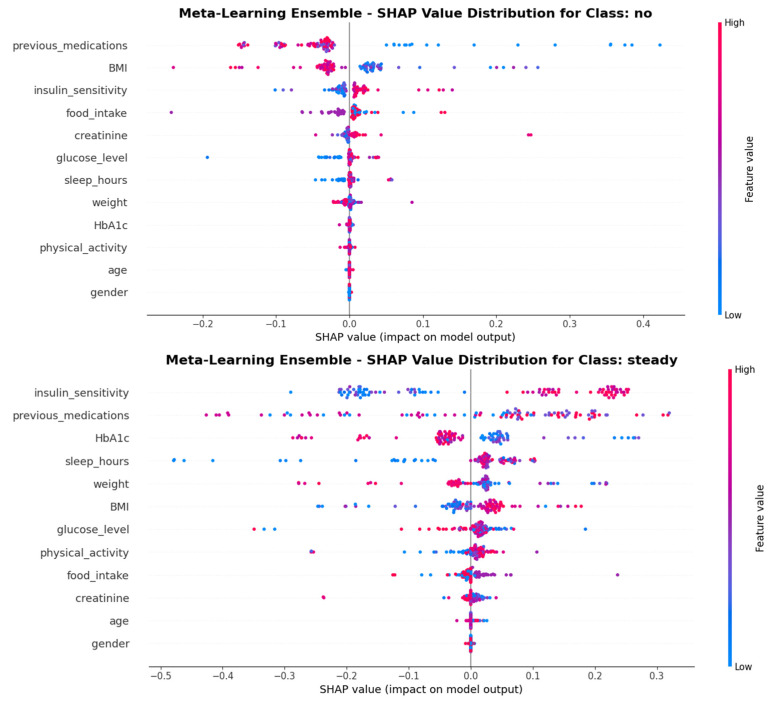

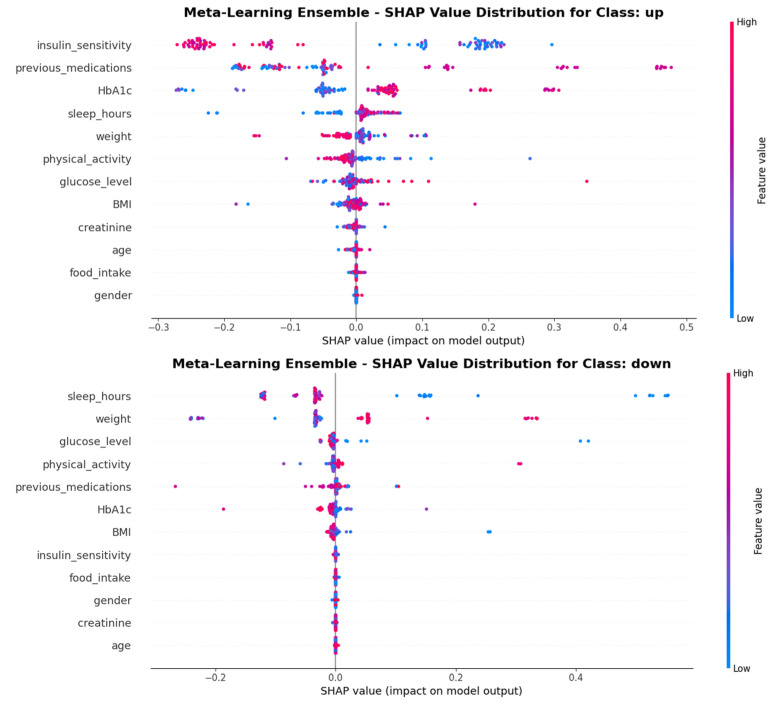
SHAP beeswarm plot showing the distribution of feature impacts on model predictions. Each point represents a single prediction. It represents the SHAP value of one feature for one sample, with color indicating feature value (red = high, blue = low) and horizontal position indicating SHAP value (positive = increases prediction probability, negative = decreases prediction probability).

SHAP analysis provided comprehensive insights into the contribution of individual features to model predictions. Figure 8 presents the global feature importance rankings based on mean absolute SHAP values, revealing that insulin sensitivity, previous medications, and sleep hours were the most influential predictors overall. The beeswarm plot (Figure 9) illustrates the distribution of SHAP values across all features, demonstrating how feature values relate to prediction impact.

Table 5 presents the top five features ranked by mean absolute SHAP values for each outcome class. For the ‘no change’ class, previous medications exhibited the highest importance (SHAP = 0.0822), followed by BMI (0.0521). Predictions for the ‘dose reduction’ class were predominantly influenced by sleep hours (SHAP = 0.1077) and weight (0.0846). The ‘dose increase’ class demonstrated the highest dependence on insulin sensitivity (SHAP = 0.1895) and previous medications (0.1466).

### 3.5. LIME Analysis and Local Interpretability

**Figure 10:**
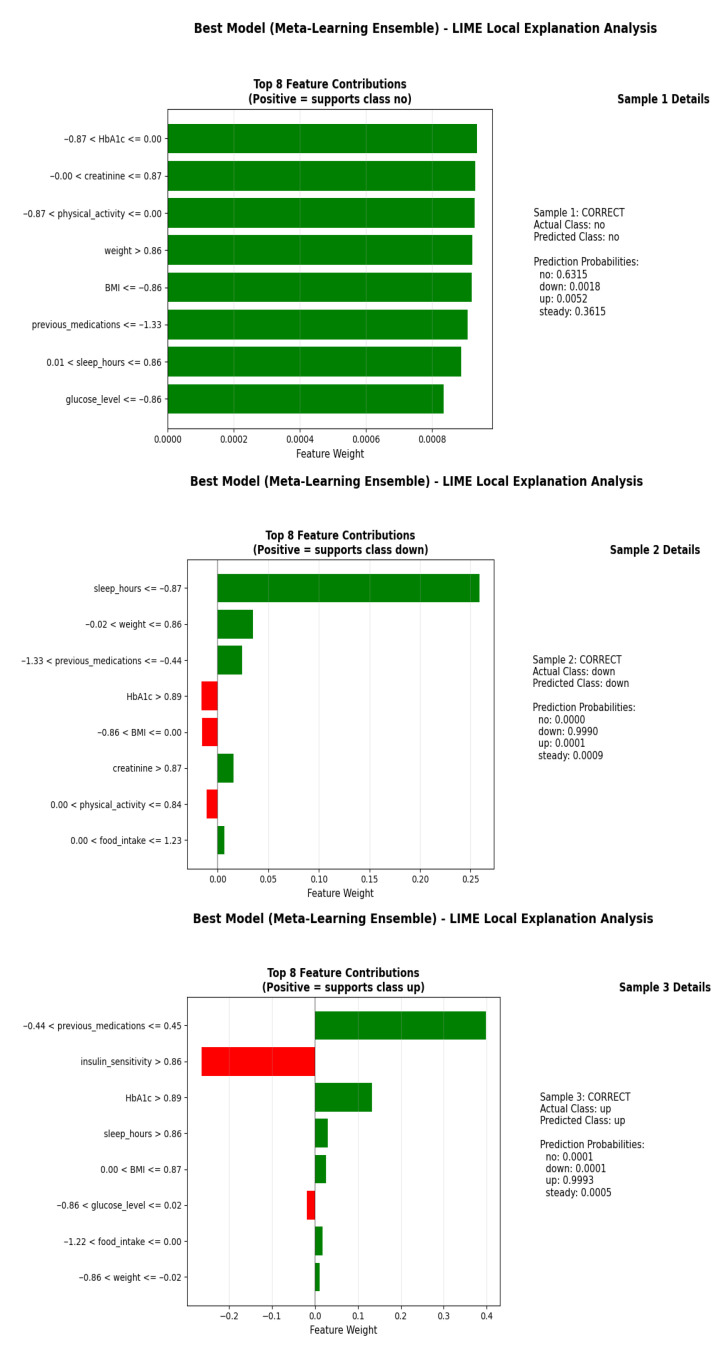
LIMEs for three correctly classified instances. Each subplot displays the top eight feature contributions for a single prediction, with bar length and direction indicating the magnitude and polarity of support for the predicted class.

LIME analysis provided detailed, instance-level insights into how individual features influenced specific predictions for the Meta-Learning Ensemble model. Unlike global methods, LIME explains each prediction separately by approximating the model locally with an interpretable surrogate. Figure 10 illustrates three example LIMEs for correctly classified instances from the testing set, each representing a different predicted class: no change, dose reduction (down), and dose increase (up).

For Sample 1 (actual class: no), the model correctly predicted no change with the highest probability (0.6315), closely followed by steady (0.3615). The LIME explanation shows that low HbA1c (≤0.00), moderate creatinine (0.00–0.87), and low physical activity (−0.87–0.00) were the strongest positive contributors to the no prediction. High weight (>0.86) and low BMI (≤−0.86) also supported no change, while very low previous medications (≤−1.33) and glucose level (≤−0.86) had weaker positive effects.

For Sample 2 (actual class: down), the model correctly predicted dose reduction (down) with near certainty (0.9990). Elevated HbA1c (>0.89) and creatinine (>0.87) were the strongest drivers, supported by moderate physical activity (0.00–0.84) and food intake (0.00–1.23). Weight and previous medication values fell in neutral-to-low ranges, contributing minimally or negatively to the prediction.

For Sample 3 (actual class: up), the model correctly predicted dose increase (up) with a probability of 0.9993. The most influential positive features were high insulin sensitivity (>0.86), elevated HbA1c (>0.89), sufficient sleep hours (>0.86), and moderate BMI (0.00–0.87). Negative contributions came from low glucose, reduced food intake, and decreased weight, which are clinically consistent with a decision to increase insulin dosage (Table 6).

LIME analysis confirmed that the model’s local decision boundaries are clinically interpretable and aligned with medical reasoning. For instance:

- High HbA1c and creatinine strongly supported dose reduction;
- High insulin sensitivity and sufficient sleep supported dose increase;
- Low HbA1c and moderate creatinine supported no change.

These local explanations enhance transparency and trust in the model by making individual predictions understandable and consistent with domain knowledge.

### 3.6. Ensemble Diversity Analysis of Base Learners

**Figure 11:**
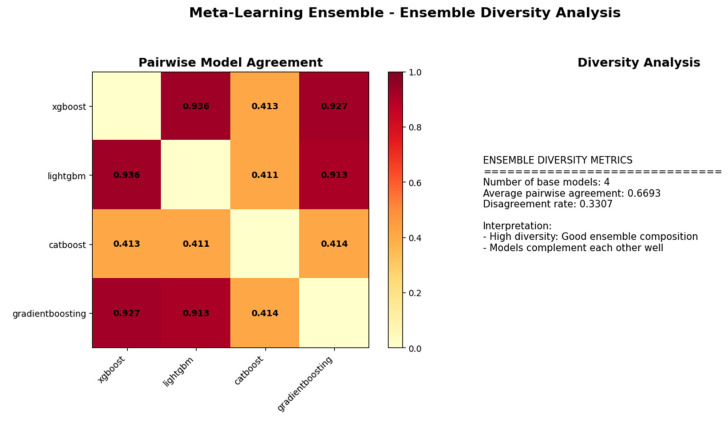
Diversity and agreement analysis of a meta-learning ensemble model.

Figure 11 illustrates the pairwise prediction agreement among four base learners—XGBoost, LightGBM, CatBoost, and Gradient Boosting within a meta-learning ensemble framework, highlighting both consensus and diversity across models. The results demonstrate a high level of agreement between XGBoost, LightGBM, and Gradient Boosting (approximately 0.91–0.94), indicating that these algorithms produce highly similar decision patterns due to their shared boosting-based architectures. In contrast, CatBoost demonstrates substantially lower agreement with the other models (around 0.41), suggesting that it captures complementary and distinct information. The average pairwise agreement of 0.6693, corresponding to a disagreement rate of 0.3307, reflects a well-balanced ensemble structure in which diversity is sufficient to reduce redundancy while maintaining predictive stability. Such a configuration is theoretically favorable for meta-learning, as the presence of heterogeneous base learners is expected to enhance generalization performance and robustness of the ensemble model.

### 3.7. Meta-Model Feature Importance Analysis

An analysis of the meta-model’s feature importance reveals that the framework relies most heavily on probability estimates generated by LightGBM, which occupies three of the top four most influential positions—specifically for predicting ‘up’ (0.2289), ‘steady’ (0.2256), and ‘no’ (0.1259) adjustments. XGBoost serves as a critical secondary contributor, particularly for identifying dose reductions (‘xgboost_prob_down’: 0.2004), where it ranks as the third most important feature overall. While Gradient Boosting and CatBoost provide more granular, auxiliary contributions at lower importance levels, the meta-learner’s prioritization of LightGBM and XGBoost suggests that these models capture the most robust decision boundaries for insulin titration.

## 4. Discussion

This study developed and evaluated an explainable meta-learning ensemble framework for predicting insulin dose adjustments in diabetic patients. Our findings demonstrate that the proposed approach achieves superior predictive performance while maintaining clinical interpretability, addressing a critical barrier to the adoption of artificial intelligence in diabetes care. This dual achievement—high accuracy paired with explainability—marks a significant step toward translating machine learning research into clinically deployable tools.

### 4.1. Principal Findings

Before interpreting the results, the clinical distinction between the four outcome classes warrants brief recapitulation. The ‘no’ class represents complete glycaemic stability requiring no dose change and no intensified monitoring, whereas the ‘steady’ class reflects a situation in which the current dose is maintained but active surveillance is indicated given the risk of future deterioration. Both the ‘up’ (dose increase) and ‘down’ (dose reduction) classes inherently require continued monitoring after any adjustment to assess treatment response and guide further titration. These distinctions carry direct implications for how class-specific performance metrics should be interpreted clinically.

The Meta-Learning Ensemble outperformed all individual classifiers and alternative ensemble configurations across multiple evaluation metrics. The achieved accuracy of 81.35% and AUC-ROC of 0.9637 represent clinically meaningful performance levels that compare favorably with recent studies on diabetes prediction.

The superior performance of the meta-learning approach over simpler ensemble strategies can be attributed to its ability to learn optimal combination weights from out-of-fold predictions, effectively capturing complex interactions between base model outputs. This finding aligns with recent work demonstrating that meta-learning architectures can adaptively weight base learners based on their reliability for specific input patterns [13,37]. This dynamic weighting is particularly advantageous in medical domains where the relationship between variables can be non-stationary or vary across patient subpopulations. The particularly high performance for the ‘dose reduction’ class holds significant clinical implications for hypoglycemia prevention. This exceptional sensitivity is critical because, in practice, the consequences of overlooking a necessary dose reduction may be far more immediately dangerous for the patient. Moreover, this model exhibits a high level of specificity (91.79%) that guarantees that patients are not exposed to unnecessary dose reduction that would otherwise result in hyperglycemic episodes. The sensitivity and specificity are balanced, which highlights the reliability of the model for clinical implementation, as both false positives and false negatives carry serious implications. The cross-entropy loss of 0.2939 also demonstrates well-calibrated probability outputs, i.e., clinicians can place confidence in the predicted probabilities when making critical decisions.

The log loss value of AdaBoost is not directly comparable with those of other models and should be interpreted not as a performance anomaly but as a structural difference in how the algorithm optimizes probability estimates. For this reason, AUC-ROC and F1 metrics were adopted as the primary indicators in model comparisons.

The 100% sensitivity observed for the ‘down’ class should be interpreted with caution. This finding likely reflects a structural characteristic of the dataset rather than clinically perfect discrimination. Specifically, the ‘down’ class appears to exhibit a markedly distinct feature distribution compared to other classes, potentially enabling the model to learn a near-perfect decision boundary within this dataset. Although all preprocessing procedures were implemented within a fold-based Pipeline framework and the test set was strictly excluded from training to prevent direct data leakage, the possibility of indirect label leakage—arising from features closely aligned with the labeling process—cannot be entirely excluded. Bootstrap resampling (n = 2000) demonstrated that this result remained stable across resamples, suggesting that it is not a random artifact of a single train/test split; however, stability within the same dataset does not guarantee real-world generalizability. Moreover, publicly available datasets may lack the variability and clinical noise inherent in routine practice, potentially amplifying separability patterns. From a clinical perspective, class-specific errors are particularly important: while missing ‘up’ cases may contribute to sustained hyperglycaemia and long-term complications, false-positive ‘down’ predictions could lead to unnecessary dose reductions and increase the risk of hypoglycaemia. Therefore, this result should be regarded as dataset-specific and requires external validation in real-world clinical cohorts before any clinical interpretation.

A more specific limitation emerges from the confusion pattern between the ‘up’ and ‘steady’ classes. Examination of the confusion matrix reveals that approximately 8.6% of patients requiring a dose increase are misclassified as ‘steady’, while approximately 7.6% of stable patients are misclassified in the opposite direction. This bidirectional confusion indicates that even the best-performing model struggles to draw a clear decision boundary between these two classes in a substantial proportion of cases, thereby constraining overall model performance. This pattern is not unexpected from a clinical standpoint: patients near the threshold between dose increase and dose maintenance are likely to share highly similar feature profiles—modestly elevated glucose levels, borderline HbA1c, and intermediate insulin sensitivity values—making the distinction between ‘up’ and ‘steady’ inherently ambiguous. The semantically adjacent nature of these two classes, combined with the dataset’s inability to capture longitudinal glycemic trends, is likely to be a key driver of this confusion. Future work should specifically address this boundary region through the use of confidence-based deferral mechanisms, clinician-in-the-loop review for borderline cases, or the incorporation of temporal glucose trajectories to improve discrimination between these clinically proximate categories.

For the model to be translatable to clinical practice, therefore, not only overall accuracy metrics but also class-specific error costs must be optimized. Future studies should aim to increase the sensitivity of the ‘up’ class and achieve a clinically acceptable error balance through the use of asymmetric cost functions or threshold optimization. In its current form, this model should be regarded not as an independent decision-maker, but as a clinician-supervised clinical decision support tool.

### 4.2. Clinical Interpretability

The integration of SHAP analysis provides transparent insights into model decision-making that align with established clinical understanding. The identification of insulin sensitivity as the predominant predictor for dose increase recommendations is physiologically coherent, as diminished insulin sensitivity directly implies a requirement for higher insulin doses to achieve equivalent glycemic effects [5]. Similarly, one study demonstrates that incorporating social network metrics into an XGBoost algorithm forms a more robust and generalizable prediction model for GDM, highlighting the significant untapped potential of social determinants in clinical risk assessment [38].

The class-specific importance patterns revealed by SHAP analysis enable clinicians to understand not merely what the model predicts, but why particular features drive specific recommendations. This transparency is essential for clinical adoption, as practitioners must be able to verify that model reasoning aligns with medical knowledge and individual patient circumstances [39,40]. The LIME findings corroborated the SHAP-level results at the instance level, with misclassified and borderline cases revealing the model’s sensitivity to feature interactions near class boundaries. This two-tier interpretability (global through SHAP, local through LIME) supports clinical auditability and may enhance adoption among healthcare teams.

### 4.3. Comparison with Previous Studies

Our results compare favorably with existing literature on ML-based diabetes prediction systems. Tasin et al. [11] achieved 81% accuracy using XGBoost with ADASYN oversampling for diabetes prediction, while Kibria et al. [41] reported 90% balanced accuracy using a weighted voting ensemble. Recent work by Houssein et al. [14] achieved exceptional performance (F1 = 0.9860) using SMOTE-ENN combined with Gradient Boosting, although this was for binary diabetes detection rather than treatment optimization. Li et al. [38] reported strong performance of boosting algorithms for diabetes-related complications, supporting the utility of gradient boosting methods in this clinical domain. Furthermore, the ability to generate both global (population-level) and local (individual patient-level) explanations allows the tool to support both clinical education and personalized, point-of-care decision-making. Notably, in contrast to numerous earlier studies, which considered binary classification [42], our work deals with a more clinically specific and challenging problem of multi-class insulin dose adjustment. This is a significant advancement from detection to management, in line with the overall transformation of digital health, which is no longer diagnosis but ongoing care optimization. Moreover, our ensemble framework also outperformed individual gradient boosting models, which suggests that multiple algorithms capture complementary patterns that a single model alone cannot identify.

This study represents a proof of concept aimed at evaluating the comparative performance and SHAP-based interpretability of different ensemble learning architectures for insulin dose adjustment prediction. While the results are promising, the clinical validity of the study is limited by its reliance on a single publicly available dataset, the inability to verify whether the labels reflect real clinical titration decisions, and the absence of a prospective design. External validation with multicenter, EHR-linked, and prospective datasets is essential before these findings can be translated to real patient care.

### 4.4. Strengths and Limitations

This study possesses several methodological strengths, including comprehensive comparison of nine ML approaches, multiple evaluation metrics capturing different aspects of classification performance, and integration of both SHAP and LIME analyses for interpretability. The use of both SHAP and LIME provides a more robust interpretability framework, as they rely on different foundational principles (coalitional game theory vs. local linear approximations), offering complementary views of model behavior. The large sample size (n = 10,000) provides statistical power for reliable performance estimation.

However, several limitations warrant consideration. The dataset originates from a single source, and external validation on independent datasets is required to confirm generalizability. The observational nature of the data precludes causal inference. Class imbalance, particularly for the minority ‘down’ class, may influence model behavior. While this imbalance was addressed through class weighting during model training rather than resampling, truly rare events in clinical practice may still be challenging to model accurately. Additionally, the study did not incorporate temporal dynamics from continuous glucose monitoring data, which could enhance prediction accuracy. Future work should also consider social determinants of health and patient-reported outcomes, which are increasingly recognized as critical factors in chronic disease management [8,12].

In this study, interpretable predictive models were developed using a comprehensive, publicly available dataset of insulin dose adjustments in diabetic patients. Therefore, further research is needed to confirm that the labels are based on actual clinical dose adjustment decisions, clinician decision rules, or long-term patient follow-up. This situation limits the clinical validity of the model, regardless of its algorithmic performance. The 100% sensitivity value for the ‘down’ class should be interpreted with caution, as it is based on a single train/test split and a class prevalence of approximately 6%. This finding should not be accepted as a generalizable result without validation on real clinical data. KernelExplainer is a method that can produce high-variance estimates and is sensitive to the choice of background dataset. It is therefore recommended that caution be exercised when interpreting KernelExplainer-based SHAP findings for global results, and that these findings be validated with larger sample sizes. As LIMEs are based on stochastic perturbation sampling, they may inherently contain instability. Without clinician validation and counterfactual testing, the clinical validity of the presented explanations is limited. No clinical expert evaluation, inter-rater agreement analysis, or counterfactual validity check was conducted for the SHAP and LIME explanations in this study. A structured evaluation protocol involving clinicians specializing in endocrinology and diabetology is planned for future work. The log loss value of AdaBoost reflects the algorithm’s probability calibration weakness in multi-class problems and may be misleading if compared directly with other models.

Publicly available datasets may contain various structural differences compared to real EHR data. Real clinical data inherently harbors natural sources of noise and variability, such as missing values, measurement errors, patient non-compliance, and individual differences in clinician decision-making. Publicly available datasets, by contrast, are often cleansed of such noise or may have been synthetically generated. This may limit the model’s robustness to the variability it will encounter in a real clinical setting. Furthermore, since it is unknown whether the dataset is representative of a specific hospital, population, or geographical region, the generalizability of the results to different clinical contexts is restricted. The findings of this study should therefore be regarded solely as methodological inferences valid for the current data structure; validation with multicenter and prospective EHR data is required for real-world generalizability.

### 4.5. Clinical Implications and Future Directions

The proposed framework has potential applications in clinical decision support systems for diabetes management. By providing interpretable recommendations based on routinely collected clinical data, the model could assist healthcare providers in optimizing insulin therapy while reducing clinician cognitive burden. Implementation considerations include integration with electronic health records, validation across diverse populations, and establishment of appropriate clinical workflows. A key step will be designing human-AI collaboration protocols that define when and how the clinician should review, accept, or override a model suggestion, ensuring the physician remains in the decision-making loop [43].

Future research should prioritize external validation, investigation of model performance in specific diabetic subgroups, and integration of additional data modalities including CGM time-series and real-world treatment adherence information. Prospective clinical trials evaluating the impact of model-assisted insulin dosing on glycemic outcomes represent an essential step toward clinical implementation. Beyond technical validation, there is a need for usability studies to assess how clinicians interact with the model’s explanations in practice. Additionally, ethical and regulatory considerations—such as liability, data privacy, and algorithmic bias—must be addressed before widespread adoption. Future versions could also explore adaptive learning, where the model continuously updates based on new patient data, and federated learning approaches to enable multi-institutional collaboration without sharing sensitive data. Ultimately, the goal is to move from a standalone prediction tool to an integrated learning health system component that improves both individual patient outcomes and population health metrics.

## 5. Conclusions

This study developed and evaluated an explainable meta-learning ensemble framework for predicting insulin dose adjustments in diabetic patients. The proposed approach achieved superior predictive performance (accuracy 81.35%, AUC-ROC 0.9637) compared to individual classifiers and alternative ensemble strategies, while maintaining clinical interpretability through comprehensive SHAP and LIME analyses. The model demonstrated particularly high sensitivity for identifying patients requiring insulin dose reduction, a clinically critical capability for hypoglycemia prevention.

Feature importance analysis revealed distinct predictor patterns across insulin adjustment categories that align with established clinical understanding, supporting the biological plausibility of model decisions. The integration of interpretability analysis facilitates clinicians’ understanding of model recommendations, supporting informed decision-making and potentially enhancing trust in AI-assisted clinical tools. This dual focus on performance and explainability directly addresses the core translational challenges of medical AI.

These findings advance the field of artificial intelligence in diabetes care by demonstrating that explainable ensemble learning approaches can effectively address the dual requirements of predictive accuracy and clinical transparency. Future work should focus on external validation, prospective clinical evaluation, and integration of additional data modalities to further enhance the clinical utility of ML/XAI-based insulin dose optimization systems.

This study is a methodological proof-of-concept aimed at comparing different ensemble learning architectures and integrating them with XAI approaches that enhance the interpretability of clinical outcomes. External validation with longitudinal prospective datasets linked to electronic health records labeled with actual titration decisions is necessary for translating the results into clinical practice. The presented work should be considered a starting point in the development of ensemble learning and interpretable predictive models and may guide the development of clinical decision support systems.

## Figures and Tables

**Table 1:** Feature Operationalization Summary.

Feature	Unit/Scale	Definition	Dataset Coding
patient_id	—	Unique identifier for each patient	Integer (not used in model)
gender	Categorical	Gender of the patient	male/female
age	Years	Age of the patient	Continuous (numeric)
family_history	Categorical	Family history of diabetes	yes/no
glucose_level	mg/dL	Blood glucose concentration	Continuous (numeric)
physical_activity	Ordinal	Physical activity level of the patient	low/medium/high
food_intake	Ordinal	Dietary intake level of the patient	low/medium/high
previous_medications	Categorical	Prior medications used by the patient	none/insulin/oral
BMI	kg/m^2^	Body Mass Index	Continuous (numeric)
HbA1c	%	Glycated haemoglobin level reflecting average glycaemia over ~3 months	Continuous (numeric)
weight	kg	Body weight of the patient	Continuous (numeric)
insulin_sensitivity	Continuous	Individual’s responsiveness to exogenous insulin	Continuous (numeric)
sleep_hours	Hours/day	Average hours of sleep per day	Continuous (numeric)
creatinine	mg/dL	Serum creatinine level reflecting renal function	Continuous (numeric)
Insulin (outcome)	Categorical	Required insulin dosage adjustment (target variable)	steady/up/down/no

**Table 2:** The optimized hyperparameter configurations for each algorithm.

Algorithm	Hyperparameters
XGBoost	{‘tree_method’: ‘hist’, ‘subsample’: 0.8, ‘reg_lambda’: 1, ‘reg_alpha’: 0, ‘n_estimators’: 400, ‘min_child_weight’: 3, ‘max_depth’: 5, ‘learning_rate’: 0.2, ‘gamma’: 0, ‘colsample_bytree’: 0.9}
LightGBM	{‘subsample’: 0.9, ‘reg_lambda’: 0.1, ‘reg_alpha’: 1, ‘num_leaves’: 20, ‘n_estimators’: 400, ‘min_child_samples’: 30, ‘max_depth’: −1, ‘learning_rate’: 0.1, ‘device’: ‘cpu’, ‘colsample_bytree’: 0.9}
AdaBoost	{‘n_estimators’: 400, ‘learning_rate’: 1.0, ‘algorithm’: ‘SAMME’}
GradientBoosting	{‘subsample’: 0.9, ‘n_estimators’: 400, ‘min_samples_split’: 10, ‘min_samples_leaf’: 1, ‘max_features’: ‘log2’, ‘max_depth’: 5, ‘learning_rate’: 0.2}
CatBoost	{‘iterations’: 100,’learning_rate’: 0.03,’depth’: 6,’random_strength’: 1, ‘bagging_temperature’: 1,’border_count’: 254,’l2_leaf_reg’: 3, ‘verbose’: 0,}

**Table 3:** Comprehensive Performance Comparison of Machine Learning Models.

Model	ACC	F1	AUC	PR-AUC	Sens	Spec	Loss
XGBoost	0.794	0.792	0.957	0.912	0.821	0.909	0.418
LightGBM	0.795	0.794	0.958	0.920	0.830	0.910	0.371
AdaBoost	0.653	0.652	0.849	0.640	0.567	0.850	10.348
GradientBoosting	0.791	0.790	0.957	0.912	0.821	0.907	0.477
CatBoost	0.793	0.793	0.958	0.916	0.802	0.908	0.373
Voting Ensemble	0.792	0.790	0.959	0.921	0.820	0.908	0.481
Stacking Ensemble	0.804	0.803	0.961	0.927	0.836	0.913	0.319
Blending Ensemble	0.798	0.798	0.949	0.907	0.821	0.911	0.825
Meta-Learning	**0.814**	**0.812**	**0.964**	**0.932**	**0.866**	**0.918**	**0.294**

ACC = Accuracy; F1 = Weighted F1-Score; AUC = Area under ROC Curve (Macro); PR-AUC = Precision-Recall AUC (Macro); Sens = Sensitivity (Average); Spec = Specificity (Average); Loss = Cross-Entropy Loss. Bold values indicate best performance.

**Table 4:** Class-Specific Performance Metrics for Meta-Learning Ensemble.

Class	Precision	Recall	F1-Score	Support
No change	0.7853	0.9143	0.8449	140
Dose reduction	0.9839	1.0000	0.9919	122
Dose increase	0.7448	0.6984	0.7209	673
Steady	0.8383	0.8516	0.8449	1065

**Table 5:** Top Five Features by Mean Absolute SHAP Value for Each Insulin Adjustment Class.

**Rank**	**No Change**	**Dose Reduction**	**Dose Increase**
1	Previous medications (0.082)	Sleep hours (0.108)	Insulin sensitivity (0.190)
2	BMI (0.052)	Weight (0.085)	Previous medications (0.147)
3	Insulin sensitivity (0.020)	Glucose level (0.016)	BMI (0.048)
4	Food intake (0.019)	Physical activity (0.012)	Creatinine (0.044)
5	Creatinine (0.011)	Previous medications (0.012)	Weight (0.043)

Values in parentheses represent mean absolute SHAP values.

**Table 6:** Summary of LIMEs for Three Example Predictions.

Sample	Actual Class	Predicted Class	Prediction Probability	Key Supporting Features
1	no	no	0.6315	Low HbA1c, Moderate creatinine, Low physical activity
2	down	down	0.9990	High HbA1c, High creatinine, Moderate physical activity
3	up	up	0.9993	High insulin sensitivity, High HbA1c, Adequate sleep

## Data Availability

The data sets used and/or analyzed during the study are available from the corresponding author on request.
